# Recent advances in materials for organic light emitting diodes

**DOI:** 10.3762/bjoc.14.168

**Published:** 2018-07-27

**Authors:** Eli Zysman-Colman

**Affiliations:** 1 Organic Semiconductor Centre, EaStCHEM School of Chemistry, University of St Andrews, North Haugh, St Andrews, Fife, KY16 9ST, UK

**Keywords:** organic light emitting diodes

Organic light emitting diodes (OLEDs) are at the cusp of becoming the dominant technology for the mobile device and display markets. This is largely due to the concerted efforts over the past thirty years to improve the material design, beginning with the first demonstrated viability of this technology in 1987 by Tang and VanSlyke [1]. Emitters in particular have undergone an evolution in design, from fluorescent compounds to phosphorescent organometallic complexes to organic thermally activated delayed fluorescence (TADF) molecules, the latter driving tremendous recent excitement within the field of organic semiconductor research. This thematic issue of the *Beilstein Journal of Organic Chemistry* covers novel phosphorescent and TADF materials design and their inclusion as emitters in OLEDs.

Some highlights in this issue include the work of Thanh-Tuân Bui et al., who provide a welcome perspective on blue TADF materials for OLEDs in the form of a review article [2]. Cristina Cebrián and Matteo Mauro review the advances made in platinum(II) complexes for OLEDs [3]. Rebecca Pittkowski and Thomas Strassner report bright blue-to-blue-green phosphorescent platinum(II) complexes employing sterically bulky diketonate ancillary ligands [4]. In addition, Lin Gan et al. describe a new molecular design approach for orange-emitting TADF molecules employing a fluorenone acceptor [5]. In the full research paper by Feng-Ming Xie et al., they disclose two bipolar, high-energy phenothiazine-5,5-dioxide-based host materials conceived to be used for deep blue OLED devices [6].

The articles in this thematic issue provide a window into the design principles used towards the development of next-generation emitter and host materials for OLEDs. I hope these articles will provide inspiration for further research in this exciting area.

Eli Zysman-Colman

St Andrews, June 2018

**Eli Zysman-Colman** obtained his Ph.D. from McGill University in 2003. He then completed two postdoctoral fellowships, one in supramolecular chemistry at the Organic Chemistry Institute, University of Zurich and the other in inorganic materials chemistry at Princeton University. After beginning his career in Canada, he moved to the University of St Andrews where he is presently Reader in Optoelectronic Materials and Fellow of the Royal Society of Chemistry. His research program focuses on the rational design of: (i) luminophores for use in organic light emitting diodes (OLEDs) and light-emitting electrochemical cells (LEECs), two types of electroluminescent devices; (ii) sensing materials employed in electro-chemiluminescence; and (iii) photocatalysts employed in photoredox catalytic reactions.
